# AN INVESTIGATION OF CDSMP OUTCOMES FOR PARTICIPANTS ENROLLING WITH DIFFERENT LEVELS OF NEED

**DOI:** 10.1093/geroni/igae098.3005

**Published:** 2024-12-31

**Authors:** Abby Teply, Christine McKibbin, Joshua Clapp, Stacy Carling, Laurance Goodwin, Catherine Carrico, Barbara Dabrowski, Elizabeth Punke

**Affiliations:** University of Wyoming, Laramie, Wyoming, United States; University of Wyoming, Laramie, Wyoming, United States; University of Wyoming, Laramie, Wyoming, United States; University of Wyoming, Laramie, Wyoming, United States; University of Wyoming, Laramie, Wyoming, United States; University of Wyoming, Laramie, Wyoming, United States; University of Wyoming, Laramie, Wyoming, United States; University of Wyoming, Laramie, Wyoming, United States

## Abstract

The Self-Management Resource Center’s evidence-based Chronic Disease Self-Management Program (CDSMP) empowers people to actively engage in their own care through a variety of evidence-based solutions. CDSMP strategies and foci align with the Age-Friendly framework - an evidence-based approach to guide health care with older adults that emphasizes 4Ms: “What Matters,” “Medication,” “Mentation,” and “Mobility.” In prior analysis of CDSMP baseline data, participants were represented by two distinct latent profiles based on age-friendly measures: individuals needing support (“NS”) and those who were coping well (“CW”). The purpose of this study was to examine differences in CDSMP outcomes among the participant profiles. Those completing baseline and follow-up assessments (n = 147) were included. Participants were predominantly White (n = 139; 94.6%) females (n = 18; 80.3%) with a mean age of 75.21 (SD = 18.83). Repeated measures ANOVAs revealed significant interactions between CW (n = 42) and NS (n = 105) groups from pre- to post-intervention. Relative to those in the CW group, those in the NS group had greater improvement on measures of interference with daily activities, F(1,144) = 5.15, p =.03, and self-efficacy, F(1,136) = 7.39, p =.01. Despite greater improvement, those in the NS profile still had greater interference with daily activities (M = 1.94, SD = 1.00) than those in the CW group (M = 1.19, SD = 1.08) at post-intervention, t(144) = 1.99, p =.05. Additional strategies may be needed to further improve outcomes for those belonging to the Age-Friendly, “needs support” profile.

